# Enhancing immune responses of ESC-based TAA cancer vaccines with a novel OMV delivery system

**DOI:** 10.1186/s12951-023-02273-8

**Published:** 2024-01-03

**Authors:** Meiling Jin, Da Huo, Jingjing Sun, Jingchu Hu, Shuzhen Liu, Mingshuo Zhan, Bao-zhong Zhang, Jian-Dong Huang

**Affiliations:** 1 Chinese Academy of Sciences (CAS) Key Laboratory of Quantitative Engineering Biology, Shenzhen Institutes of Advanced Technology, Shenzhen Institute of Synthetic Biology, Chinese Academy of Sciences, Shenzhen, China; 2 School of Biomedical Sciences, Faculty of Medicine, Li Ka Shing, The University of Hong Kong, Pokfulam, Hong Kong SAR, China; 3 Department of Clinical Oncology, Shenzhen Key Laboratory for Cancer Metastasis and Personalized Therapy, The University of Hong Kong-Shenzhen Hospital, Shenzhen, China; 4 Guangdong-Hong Kong Joint Laboratory for RNA Medicine, Sun Yat-Sen University, Guangzhou, 510120 China

**Keywords:** Embryonic stem cell, Epitopes, Tumor immunity, OMVs, Vaccines

## Abstract

Embryonic stem cell (ESC)-derived epitopes can act as therapeutic tumor vaccines against different types of tumors Jin (Adv Healthc Mater 2023). However, these epitopes have poor immunogenicity and stimulate insufficient CD8^+^ T cell responses, which motivated us to develop a new method to deliver and enhance their effectiveness. Bacterial outer membrane vesicles (OMVs) can serve as immunoadjuvants and act as a delivery vector for tumor antigens. In the current study, we engineered a new OMV platform for the co-delivery of ESC-derived tumor antigens and immune checkpoint inhibitors (PD-L1 antibody). An engineered *Staphylococcal* Protein A (SpA) was created to non-specifically bind to anti-PD-L1 antibody. SpyCatcher (SpC) and SpA were fused into the cell outer membrane protein OmpA to capture SpyTag-attached peptides and PD-L1 antibody, respectively. The modified OMV was able to efficiently conjugate with ESC-derived TAAs and PD-L1 antibody (SpC-OMVs + SpT-peptides + anti-PD-L1), increasing the residence time of TAAs in the body. The results showed that the combination therapy of ESC-based TAAs and PD-L1 antibody delivered by OMV had significant inhibitory effects in mouse tumor model. Specifically, it was effective in reducing tumor growth by enhancing IFN-γ-CD8^+^ T cell responses and increasing the number of CD8^+^ memory cells and antigen-specific T cells. Overall, the new OMV delivery system is a versatile platform that can enhance the immune responses of ESC-based TAA cancer vaccines.

**Graphical Abstract:**

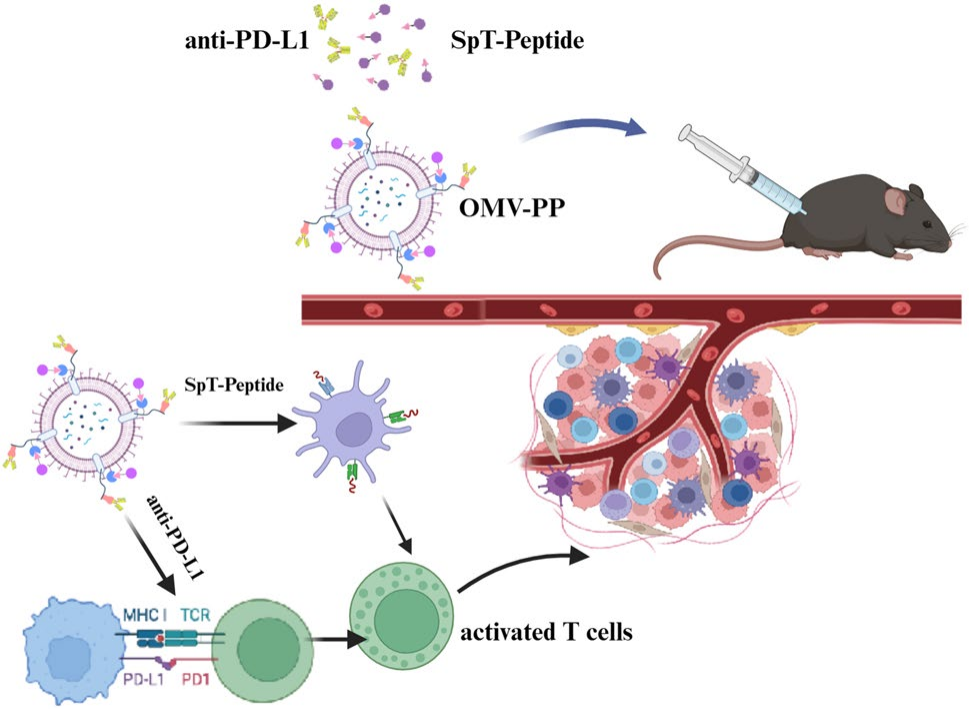

**Supplementary Information:**

The online version contains supplementary material available at 10.1186/s12951-023-02273-8.

## Introduction

Immunotherapies have been gaining increasing attention and taking the spotlight away from conventional treatment methods [1, 2]. Cancer vaccines have the potential to target a broader set of intracellular antigens and to prime tumor-reactive T cells [3]. Recent research has indicated that the gene expression pattern of embryonic stem cells (ESCs) and induced pluripotent stem cells (iPSCs) have much in common with various types of cancer cells [4]. Mice pre-immunized with irradiated iPSCs or ESCs induced tumor antigen-specific T cells against various types of tumors [4, 5]. Moreover, ESCs or iPSCs can express tumor-associated antigens (TAAs) that contain MHC class I or MHC class II epitopes, which could potentially be developed into cancer vaccines. In our previous study, several of these expressed TAAs were identified to have great immunotherapy potential against bladder cancer [1]. Here, we further evaluated the therapeutic effects of these antigens as a universal tumor vaccine.

Programmed cell death 1 ligand 1 (PD-L1), a transmembrane glycoprotein expressed by both immune cells and cancer cells, belongs to the immune checkpoint pathway. It serves as a pro-tumorigenic factor and suppresses the immune response [6, 7]. Immune checkpoint blockade of PD-L1 can be used treat ‘inflamed’ cancers that have been infiltrated by previously primed tumor-reactive T cells [8]. However, only a minority of cancer patients benefit from immune checkpoint blockade treatment [9]. Although the reason for treatment failure is complex, it is related to the insufficient numbers of T cells or poorly functioning pre-existing T cells (due to epigenetic dysfunction or the acquisition of other immunosuppressive signals), disruption of antigen presentation that leads to decreased T cell recognition of the tumor, primary resistance to IFN-γ signaling, and the immunosuppressive tumor microenvironment [3, 10] Long-term use of PD-L1 inhibitors can also lead to the occurrence of immune-related adverse events such as humoral autoimmunity [11]. These issues have prompted the need for new strategies to boost the antitumor T cell response, such as the development of more effective immunogenic therapeutic cancer vaccines. Here, we tested the combined treatment of immune checkpoint blockade with ESC-derived TAAs as an antitumor therapeutic. In previous studies, many of the explored cancer vaccine formulations did not elicit sufficient tumor antigen-specific CD8^+^ T cell responses, which are crucial for repressing the tumor [12]. This is mostly attributed to several biological barriers, including low levels of antigen cross-presentation, insufficient T cell activation, and the immunosuppressive tumor microenvironment [13]. These challenges have stimulated the development of a wide range of materials-based strategies (e.g., nanoparticles, microparticles, scaffolds, hydrogels) for the delivery of cancer vaccines.

The current research aims to use outer membrane vesicles (OMVs) as the platform for the co-delivery of TAAs and PD-L1 antibodies for treating cancer. The OMVs are membrane-derived nano-scale particles with a size of 30–250 nm, which are secreted by Gram-negative bacteria and are vital for bacterial homeostasis and communication [14]. The surface of OMVs express particular antigens and together with their innate composition could be exploited as potential pathogenic adjuvants against antecedent bacterial infection such as gonococcus [15]. Given its plasticity, much research has been done to modify the ability of OMVs to carry homologous and heterologous antigens, while attenuating the toxicity [16]. Some advantages of OMVs that make them ideal cancer vaccine vectors include its nano-sized vesicles that maintain the integrity of antigens and its potent immunogenicity that enhances the host immune response. Modified or engineered OMVs have already been used directly as cancer vaccines [14] or to deliver tumor vaccines that induce strong IFN-γ- and T cell-mediated antitumor response, which suggest OMVs have much potential as immunotherapeutic agents [17, 18]. For instance, engineered OMVs expressing human papillomavirus type 16 early protein E7 (HPV16E7) were able to inhibit tumor growth in mice bearing lung cancer [19]. However, vaccines developed for human cancers require the rapid and specific presentation of antigens originating from the tumors. To meet this challenge, we engineered OMVs using the SpyCatcher (SpC)-SpyTag (SpT) system.

The SpC-SpT system consists of a 135-residue domain and a 13-residue peptide, both of which originate from the CnaB2 domain of FbaB, a microbial surface component recognizing adhesive matrix molecule (MSCRAMM) from *Streptococcus pyogenes* [20–22]. Here, we first inserted the coding gene of SpC into the sequence of outer membrane protein A (OmpA) on OMVs. Upon introduction of the SpT component, a strong isopeptide bond is rapidly formed between SpC and SpT. Different antigens can be easily captured and conveyed on modified SpT. To increase the antitumor effect of the tumor vaccines, we designed the new platform to also deliver anti-PD-L1 or anti-PD-1 antibodies to modulate the immunosuppressive tumor microenvironment. Blocking the interaction between PD-1 and PD-L1 can boost the immune response against cancer cells [23]. We engineered *Staphylococcal* Protein A (SpA), a protein capable of non-specifically binding to immunoglobulins, and also fused it with OmpA on OMVs. Consequently, the SpT/SpC/SpA system was able to display both ESC-derived TAAs and PD-L1 antibodies on the surface of OMVs (OMV-PP). The results showed that our engineered OMV system was able to co-deliver synthetic long peptide antigens containing ESC-derived epitopes and anti-PD-L1 antibodies to the tumor. The OMV-PP was able to enhance the CD8^+^ T cell responses to a diverse array of TAAs and dramatically improve immune checkpoint blockade in murine tumor models.

## Methods

### Materials

Peptides with a tag (tag sequence: GGGAHIVMVDAYKPTK) were synthesized by GL Biochem (Shanghai, China). Dulbecco’s Modified Eagle (DMEM) Medium, Roswell Park Memorial Institute (RPMI) 1640 medium, fetal bovine serum (FBS), and penicillin–streptomycin (PS) were obtained from ThermoFisher Scientific (Waltham, MA, USA). Enzyme-linked immunospot (ELISPOT) assay kit was purchased from MabTech (Nacka Strand, Sweden). Please refer to Table 1 for other reagents and antibodies.

**Table 1:** Chemicals, peptides and Recombinant Proteins

	Reagent or resource antibody	Source	Identifier
1	PE/Cy7 anti-mouse CD3	Biolegend	100220
2	APC anti-mouse CD4	Biolegend	100412
3	Alexa Fluor^®^ 700 anti-mouse CD25	Biolegend	102024
4	PerCP/Cyanine5.5 anti-mouse CD8a	Biolegend	100733
5	Pacific Blue™ anti-mouse/human CD44	Biolegend	103020
6	Brilliant Violet 605™ anti-mouse CD45	Biolegend	103139
7	PE/Dazzle™ 594 anti-human/mouse Granzyme B Recombinant	Biolegend	372207
8	Alexa Fluor^®^ 488 anti-mouse FOXP3	Biolegend	126405
9	PE anti-mouse H-2Dk	Biolegend	110307
10	Brilliant Violet 421™ anti-mouse F4/80	Biolegend	123131
11	PE/Dazzle™ 594 anti-mouse I-A/I-E	Biolegend	107647
12	FITC anti-mouse CD86	Biolegend	105005
13	PE/Cy7 anti-mouse/human CD11b	Biolegend	101215
14	APC anti-mouse CD11c	Biolegend	117309
15	PerCP/Cyanine5.5 anti-mouse CD49b (pan-NK cells)	Biolegend	108915
16	Alexa Fluor^®^ 700 anti-mouse Ly-6G/Ly-6C (Gr-1)	Biolegend	108421
17	PE/Cy7 Rat IgG2b, κ Isotype Ctrl	Biolegend	400617
18	APC Rat IgG2b, κ Isotype Ctrl	Biolegend	400611
19	PerCP/Cyanine5.5 Rat IgG2a, κ Isotype Ctrl	Biolegend	400531
20	Pacific Blue™ Rat IgG2b, κ Isotype Ctrl	Biolegend	400627
21	Brilliant Violet 605™ Rat IgG2b, κ Isotype Ctrl	Biolegend	400657
22	Alexa Fluor^®^ 488 Rat IgG2b, κ Isotype Ctrl	Biolegend	400625
23	PE Mouse IgG2a, κ Isotype Ctrl	Biolegend	400211
24	Brilliant Violet 421™ Rat IgG2a, κ Isotype Ctrl	Biolegend	400549
25	PE/Dazzle™ 594 Rat IgG2b, κ Isotype Ctrl	Biolegend	400659
26	FITC Rat IgG2a, κ Isotype Ctrl	Biolegend	400505
27	Alexa Fluor^®^ 700 Rat IgG2b, κ Isotype Ctrl	Biolegend	400628
28	TruStain fcX™ (anti-mouse CD16/32)	Biolegend	101320
29	True-Nuclear™ Transcription Factor Buffer Set	Biolegend	424401

1	Penicillin–Streptomycin-Glutamine (100X)	Gibco	10378016
2	FBS	GIBCO	10099–141
5	DMEM	GIBCO	10566–016
6	Opti-MEM	Gibco	31985070
7	Trypsin–EDTA (0.25%), phenol red	Gibco	25200072
8	InVivoMab anti-mouse CD4	BioXcell	BE0003-1-5MG
9	InVivoMab anti-mouse CD8	BioXcell	BE0004-1-5MG
10	100um cell strainer	Falcon	352360
11	70um cell strainer	Falcon	352350
12	Percoll	GE Health	17–0891-02
13	DPBS	Hyclone	SH30028.02
14	TLRL-1826 ODN	Invivogen	tlrl-1826–1
15	Dimethyl sulfoxide (DMSO)	Sigma-Aldrich	D2650
16	Collagenase A	Roche	10103586001
17	HBSS	Servicebio	G4204-500
18	10XPBS	Servicebio	G4207-500
19	mouse IFN-gamma elispot plus(plus) HRP	MabTech	3321-4HST-2
20	Bovine Serum Albumin	SIGMA	V900933-100G
21	mouse IFN-gamma elispot plus(plus) HRP	MabTech	3321-4HST-2
22	CD45 MicroBeads, mouse	Miltenyi Biotec	130–052-301
23	Mouse IFN-gamma ELISpot PLUS (HRP), strips	MabTech	3321-4HST-10
24	EmbryoMax™ Nucleosides (100X)	merck	ES-008

### Bacterial strains and media

Bacterial strains and plasmids are listed in Table 2. *E. coli* MG1655 wild type (WT) strain was used as the parental strain for isogenic gene deletion and chromosomal modification. *E. coli* strain BL21 (DE3) was used for recombinant protein expression. Standard Luria–Bertani (LB) broth with appropriate antibiotics (100 mg/mL ampicillin, 50 mg/mL kanamycin, or 25 mg/mL chloramphenicol) was used for *E. coli* culture.

**Table 2:** Bacterial strains and plasmids used in this study

Strain Number	Genotype/Phenotype	reference/Source
MG1655	Wild type* Escherichia coli*	Laboratory stock
JS09	MG1655 Δ*lpxM*	https://doi.org/10.3389/fimmu.2023.1088501
JS26	MG1655 Δ*lpxM*Δ*pal*	This study
JS86	MG1655 Δ*lpxM*Δ*pal ompA-spycatcher-spA::kan*	This study

### Cell lines and cell culture

The MB49 bladder cancer cell line (SCC148, Sigma-Aldrich, St. Louis, MO, USA) and LLC lung cancer cell line (CRL-1642, ATCC, Virginia, USA) were grown in DMEM with 10% FBS and 1% PS in a humidified atmosphere at 37 °C and 5% CO2.

### Bacterial OMV preparation and characterization

Bacteria were cultivated in LB broth supplemented with 1 mg/mL glucose and 50 µg/mL kanamycin overnight at 37 °C with shaking (220 rpm), followed by centrifugation at 4000 rpm for 20 min at 4 °C. The supernatant was filtered in a 0.45-µm filter (Millipore, Merck, Germany) and centrifuged at 150000 g for 1 h at 4 °C. The pellets containing OMVs were resuspended in Dulbecco’s phosphate buffered saline (DPBS) and filtered in a 0.22-µm filter (Millipore).The obtained OMVs were stored at -80 °C until further use. The total protein concentration of the prepared OMVs was determined by bicinchoninic acid assay. Transmission electron microscopy was used to examine the morphology of the prepared OMVs.

### SpyCatcher and SpyTag, SpA and IgG reaction assay

Equal amount (50 μg) of OMV-SpyCatcher-SpA, SpyTag-peptides and 0.5 μg anti-PD-L1 were incubated at room temperature for 30 min. Unbound SpyTag-GFP, SpyTag-mCherry and IgG in the reaction were removed by ultrafiltration using a 0.5 mL 100-KDa ultrafiltration unit (Millipore). For the first round of ultrafiltration, the reaction was supplemented to a volume of 300 μl in total by adding DPBS and then centrifuged at 14,000 g until a volume of 50 μl. The resultant reaction was resuspended to 300 μl for the second round of ultrafiltration to a volume of 50 μl. To tested OMV-SpA-anti-PD-L1 targeted to tumor cells, 50 μg OMV-SpyCatcher-SpA-anti-PD-L1 were incubated with MB49 cells at room temperature for 30 min. The cells were washed three times in PBS, and subjected to flow cytometry analysis using FITC channel (CytoFLEX, Beckman). To perform the reaction assay in living cells, ΔlpxM and ΔlpxM ompA-spycatcher bacterial cells were collected and washed in PBS. Bacteria were then adjusted to OD600 of 0.3 and incubated with or without 100 μg Spytag-GFP or Spytag-mCherry or 0.5 μg IgG-antibody (PE-cy7) at room temperature for 10 min. Samples were washed three times with ice-cold PBS and subjected to flow cytometry analysis using FITC channel, APC channel and PE-cy7 channel (CytoFLEX, Beckman).

### Tumor therapy and immunization experiments

Male C57BL/6 mice (6–8 weeks old) were treated with MB49 or LLC cancer cells (3 × 10^5^) by subcutaneous injection in the lower back. For OMV linked TAAs and anti-PD-L1 group, 50 μg OMV-SpyCatcher were incubated with TAAs (100 μg each) and anti-PD-L1 (0.5 μg each) for 30 min. 50 μg empty OMVs and PBS were also injected as control groups. Subsequently, animals were divided into groups and immunized with different formulations by subcutaneous injection into the upper back. Tumor size was measured every 3 days. On day 5 after the last immunization, mice were sacrificed and blood, tumor, spleen, and draining lymph nodes (dLNs) were harvested. Blood samples were collected in sterile 1.5 mL microtubes and anticoagulant tubes to obtain serum and plasma, respectively. Animal protocols were approved by the Institutional Animal Care and Use Committee (IACUC), Shenzhen Institute of Advanced Technology (SIAT), Chinese Academy of Science.

### Isolation of lymphocytes from spleens, dLNs, and tumors

Spleens were gently mashed using a 2-mL syringe plunger, filtered through a 70-μm strainer (Falcon, Corning, Germany), and then suspended in 1640 medium, followed by centrifugation to obtain splenocytes. After adding erythrocyte lysis buffer and washing in PBS, cells were collected for ELISPOT assay or stored at − 80 °C until further use. The dLNs were processed as described above to acquire lymphocytes. Tumor tissues were ground by a pestle and cultured in RPMI medium with FBS, collagenase, DNase, and 4-(2-hydroxyethyl)-1-piperazineethanesulfonic acid (HEPES). After shaking at 37 °C for 20 min, the samples were filtered through a 100-μm strainer and suspended in percoll gradient (General Electric Healthcare, USA) to remove non-immune cells. Next, ammonium chloride potassium (ACK) lysis buffer was used to remove red blood cells. After washing with PBS, tumor-infiltrating leukocytes (TILs) were collected for ELISPOT assay or stored at -80 °C for subsequent analyses.

### ELISPOT assay

Lymphocytes derived from spleens or TILs (5 × 10^5^ cells) were co-cultured with different peptides for 25 h to detect the secretion of IFN-γ by ELISPOT assay according to the manufacturer’s instructions (MabTech, Nacka Strand, Sweden). The size and number of IFN-γ-positive spots were calculated using Adobe Photoshop CS6 software.

### Staining of inflammatory cells for FACS analysis

Peripheral blood mononuclear cells (PMBCs) were obtained from blood and suspended in 100 μL FACS buffer containing DPBS and 2% FBS. The PBMCs and lymphocytes derived from dLNs were blocked using an FcR-blocking Reagent (BioLegend, San Diego, CA, USA) and divided into two groups. One group was stained with a surface marker panel containing CD3, CD4, CD25, CD8, CD44, CD45 (BioLegend), and intracellular markers Granzyme-B and FoxP3 (BioLegend). The other group was stained for F4/80, MHC-II, CD86, CD11b, CD11c, NK1.1, Ly6-G, and CD45 (BioLegend). Rat IgG2b isotype control (BioLegend) was used for CD3, CD11b, CD4, CD11c, CD25, Gr-1, CD44, CD45, Foxp3, and Granzyme B; rat IgG2a isotype control (BioLegend) for CD86 and F4/80, CD8a and NK1.1; and rat IgG1 isotype control (BioLegend) for MHC II. Staining with intracellular markers required fixation and permeabilization of cells following extracellular staining. Samples were analyzed on a Beckman Flow Cytometer in the Beckmann FACS facility.

### Multiplex cytokine assay

Levels of IL-2, IL-4, IL-6, and IFN-γ were detected in the serum by flow cytometry using Cytometric Bead Array (CBA) Flex Set (BD Biosciences, USA).

### Biodistribution of OMVs

Male C57BL/6 mice (6–8 weeks old) bearing MB49 tumors were divided into three groups. On day 20 after tumor inoculation, mice were treated with different OMV formulations by subcutaneous injection in the upper back on days 1, 2, 4, or 6. In vivo optical imaging (IVIS) system (Caliper Spectrum IVIS, IV, Caliper, USA) was used to monitor the distribution of OMVs in mice.

### In Vivo T lymphocyte subset depletion

Anti-CD4 monoclonal antibody (mAb) (10 mg kg^−1^, clone GK1.5, Bio X Cell, Lebanon, USA) and anti-CD8 mAb (10 mg kg^−1^, clone 53–6.7, Bio X Cell) were used for the deletion of CD4^+^ and CD8^+^ T cells, respectively. Mice were administered the antibodies as described above on days -1, 1, and 3 after tumor inoculation, and then vaccinated every 3 days for a total of five times. Mice were euthanized when the tumor size reached 1500 mm^3^, and survival time was recorded and analyzed by the Kaplan–Meier method. Log-rank test was used to calculate the P-value.

### Selection of upregulated genes shared by tumors and stem cells

Gene expression data from normal tissue and ESCs (C57) from C57BL/6 mice were downloaded from BgeeDB (version 14.0) [24]. The RNA-seq data of three tumor cell lines (LLC, MB49, and Hepa1-6) and ESCs (129) were processed accordingly, and two expression measurements (raw count and TPM) were obtained using kallisto [25]. Gene expression analysis was performed on the three mouse tumor cell lines and two ESC cell lines, and on multiple selected normal tissues by DESeq2 [26] using gene raw counts as the input with default parameters. Genes with adjusted P-value < 0.005 and log2 fold change > 3 were considered to be significant. The top 100 significantly upregulated genes in tumors across multiple cancer types from The Cancer Genome Atlas (TCGA) were retained. The differential expression results were retrieved from OncoDB [27] with a cutoff of a > twofold change and an adjusted P-value of > 0.05 for significantly upregulated genes, resulting in more than 12 cancer types retained.

### Processing the sequencing data

The RNA-seq data were used for gene expression quantification using Salmon [28]. Differential gene expression analysis was carried out between PBS and OMV-PP groups by DESeq2. ClusterProfiler was used for gene ontology enrichment analysis [29], similar GO terms were merged by the “simplify” function with a cutoff of 0.65. Cell type functional enrichment was conducted in xCell [30] using a mouse gene expression profile. Immune sequencing data was used in the T cell receptor repertoire analysis conducted in MiXCR. Samples with observed clone numbers of < 30 were excluded from the downstream analysis. Productive T cell receptor β gene was used for T cell immune repertoire diversity estimation. Metrics including Shannon–Wiener diversity and Simpson index were computed by MiXCR after downsampling to the unification number of UMI (unique molecular identifiers) clones.

### Quantification and statistical analyses

All values in bar graphs and curves are expressed as mean ± SD. Intergroup differences were appropriately assessed by either unpaired two-tailed Student’s t-test or one-way analysis of variance (ANOVA) with multiple comparison tests using PRISM GraphPad software. ∗ P < 0.05, ∗  ∗ P < 0.01, ∗  ∗  ∗ P < 0.001, ∗  ∗  ∗  ∗ P < 0.0001.

## Results

### Preparation and characterization of the OMV dual delivery system

**Fig. 1:**
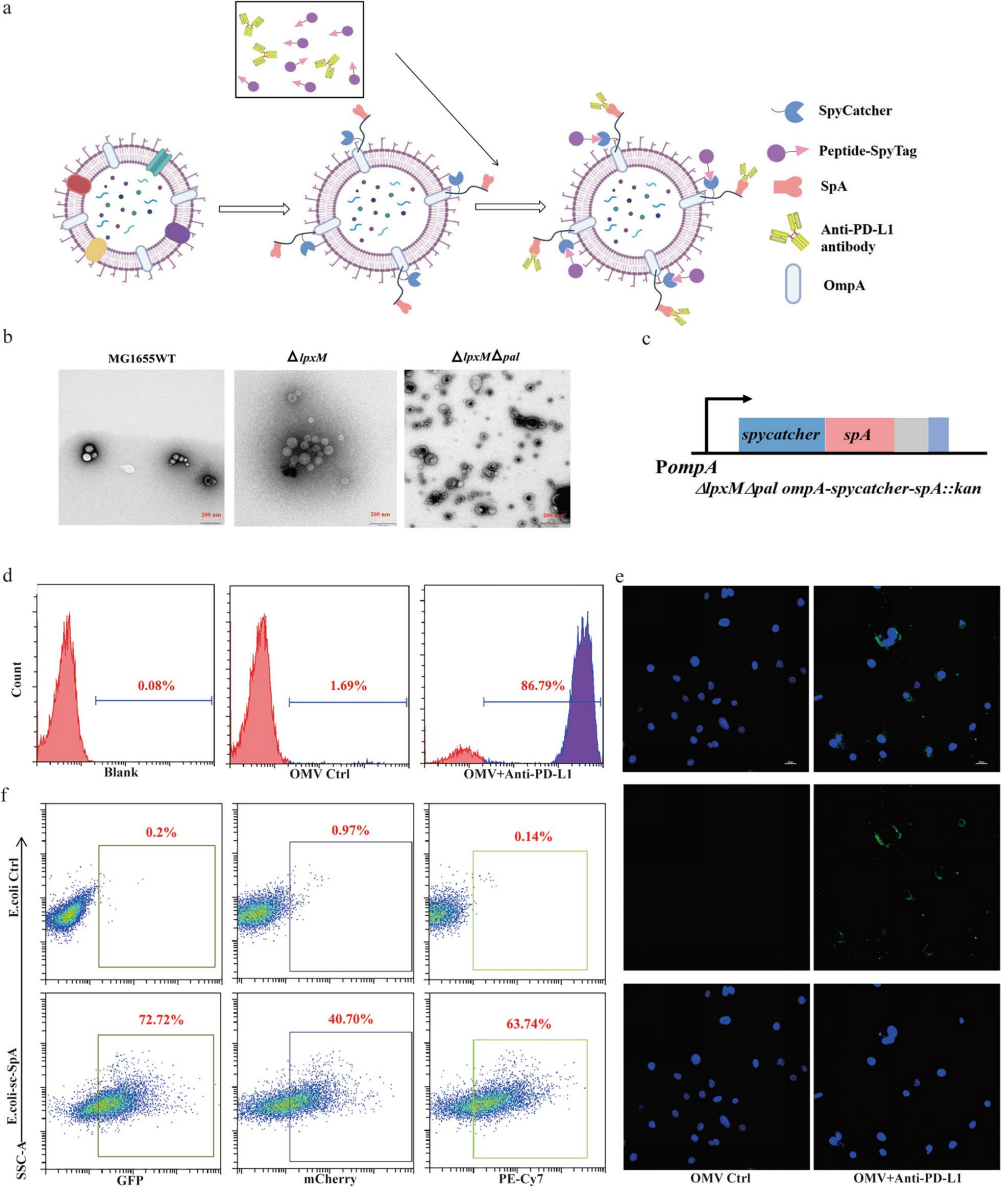
Generation and characterization of OMV-PP. **a** Schematic illustration of the OMV-based delivery system. The OMVs extracted from Δ*lpxM*Δ*pal* strain were bound with both SpT-attached TAAs and PD-L1 antibody. **b** Representative TEM image of OMVs. Scale bar: 200 nm. **c** Sketch map of Δ*lpxM*Δ*pal ompA-spC-spA* (the drawing is not to scale). **d**, **e** Examination of the ability of OMV-SpA to capture PD-L1 antibody. d) Flow cytometry analysis and **e** confocal microscopy images (blue, DAPI; green, FITC). Scale bar: 70 μm. **f** Testing the capability of OMV-SpC-SpA to capture SpT-attached mCherry, GFP, and PE-Cy7-labeled IgG antibody

To design a viable system for the co-delivery of TAAs and antibodies, we engineered OMVs to express SpC and SpA in its surface ompA (Fig. 1a). The Δ*lpxM*Δ*pal* strain was created using MG1655 Δ*lpxM* [31] to further delete the *pal* gene, as previously described [31]. Briefly, PCR-amplified DNA fragments containing 40 bp homologous arms flanking the *pal* open reading frame together with a selection antibiotic marker were electroporated into MG1655 Δ*lpxM* harboring lambda red recombinase expression plasmid pKD46. Positive clones were selected with 25 mg/mL chloramphenicol and verified by DNA sequencing. Subsequently, the antibiotic selection marker flanked by the loxP sites was removed using a helper plasmid p705Cre. Transmission electron microscopy images showed the knockout of *lpxM* and *pal* genes did not affect the morphology and size of OMVs (Fig. 1b, Additional file 1: S1b). The Δ*lpxM*Δ*pal ompA-SpC-SpA* strain was further constructed using a similar recombineering method. An *ompA(1–393 bp)-G4S linker-SpC-SpA-6his* fusion gene was synthesized and used to replace the chromosomal *ompA* gene (Fig. 1c). Positive clones were selected using 50 mg/mL kanamycin and confirmed by DNA sequencing. Primers used in this study are listed in Table 3. The ability of the engineered bacterial OMVs to capture PD-L1 antibody was determined by the binding efficiency measured by flow cytometry. The results showed the PD-L1 antibody was captured with high efficiency by SpA on the surface of engineered OMVs (Fig. 1d). The modified OMVs were then extracted and analyzed by Western blotting to detect SpC fused to OmpA. As shown in Additional file 1: Figure S1a, SpC and OmpA were both detected in isolated OMVs. Next, the engineered OMVs were mixed with PD-L1-FITC antibody and co-cultured with PD-L1^+^ MB49 cells. The assays demonstrated that SpA was successfully linked to OMVs (Fig. 1e). Subsequently, we examined the capture efficiency of SpC and SpA on the surface of bacteria. The results demonstrated that the engineered strain effectively captured SpT-linked GFP and mCherry, and PE-Cy7-labeled IgG antibodies (Fig. 1f). The engineered bacteria secreting the OMVs were used in the following studies.

**Table 3:** Primers used in this study

Primers	Sequences
delpal-1	TGTGATAATAATTAATTGAATAGTAAAGGAATCATTGAAGCCGATCATATTCAATAACC
delpal-2	CAATAGTTGATGTCTGAAGTTACTGCTCATGCAATTCTCAAGCTTATCGATACCGTCGA
C-pal-1	GATCCGTGCTGAATTTGGTT
C-pal-2	AAGTTGAGTGACGCGGTCTT
ompASCspA-1	GGGTGGCATGGTATGGCGTGCAGACACTAAATCCAACGTTGGCGGCGGCGGCAGCGGCGC
ompASCspA-2	AAAACCCCGCAGCAGCGGGGTTTTTCTACCAGACGAGAACTGTAGGCTGGAGCTGCTTCGA
C-ompA-1	GTTTCCGCGATTCTCTTCTG
C-ompA-2	TAATGCGGAACACCAGCATA

### Delivery of TAA-modified OMVs to tumors and attenuation of tumor growth

**Fig. 2:**
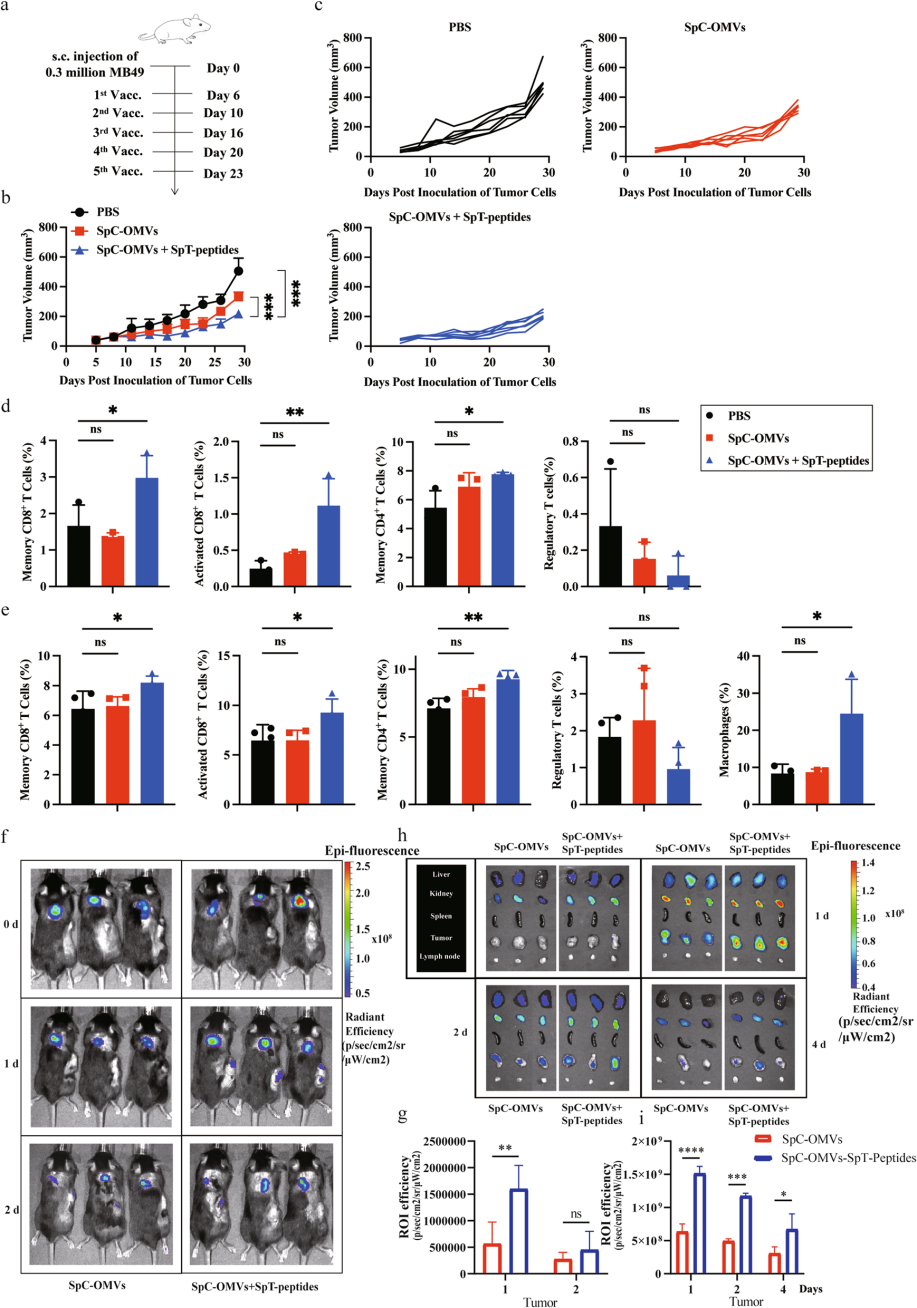
TAA-bound OMVs specifically targeted tumors and repressed bladder cancer growth. **a** Schematic illustration examining how OMV-P inhibits bladder cancer growth. C57BL/6 mice were s.c. injected with MB49 cells in the right flank on day 0 and s.c. immunized with PBS, OMV, or OMV-P on days 6, 10, 16, 20 and 23. **b**, **c** Antitumor effect of OMV-P. Tumor size was measured every 3 days. Quantification of the tumor size data are presented in (**b**, **c**). Experiments were repeated three times. d) Quantitative analysis of different subsets of CD3^+^CD45^+^ T cells in lymph nodes by flow cytometry (n = 3). Memory CD4^+^ T cells, CD4^+^CD44^+^ T cells. Memory CD8^+^ T cells, CD8^+^CD44^+^ T cells. Activated CD8^+^ T cells, CD8^+^Granzyme-B^+^ T cells. Treg, CD4^+^CD25^+^FoxP3^+^ T cells. e) Quantitative analysis of different subsets of immune cells in PBMCs by flow cytometry (n = 4 or 3). Memory CD4^+^ T cells, memory CD8^+^ T cells, activated CD8^+^ T cells, and Treg were analyzed as described above (n = 4). Macrophages (n = 3), CD45^+^CD11b^+^F4/80^+^ cells. f, h) Cy7-labeled OMV-P were systemically injected into C57/BL6J mice bearing MB49 tumor cells. Whole body distribution of the injected Cy7-OMV-P was observed by an in vivo imaging system on days 0, 1, and 2 after injection. Liver, kidney, spleen, tumor, and lymph node were isolated to measure the accumulation of Cy7 fluorescence in different organs. d, day. **g**, **i** Quantification of fluorescence in the tumors (in vivo or ex vivo) (n = 3). Data are shown as mean ± SD. One-way ANOVA with a Tukey multiple comparisons test. NS, no significance; *, p < 0.05; **, p < 0.01; ***, p < 0.001; ****, p < 0.0001

In our previous study, we verified the tumor inhibitory effect of ESC-derived TAAs on bladder cancer^11^. To investigate the effectiveness of our new delivery system, we synthesized peptides according to the amino sequences of the selected TAAs. Mice bearing MB49 tumor were immunized five times with OMVs with SpT-peptides (OMV-P) or OMVs or PBS (Fig. 2a). The peptide-modified OMV-P significantly repressed bladder cancer growth (Fig. 2b, c). On day 29 after tumor inoculation, mice were sacrificed and blood and dLNs were collect for flow cytometry analysis. The results showed the mice primed with OMV-P had increased levels of CD8^+^ effector T cells, CD4^+^ memory T cells, and CD8^+^ memory T cells in PBMCs and dLN-derived lymphocytes (Fig. 2d, e). Besides the altered lymphocytes, we also found augmented macrophages in OMV-P-treated mice.

To understand the underlying mechanism of the induced antitumor effects of OMV-P, we labeled OMVs with Cy7 and tracked its distribution by in vivo imaging after systemic administration. Fluorescence intensity was measure at 24 and 48 h after the subcutaneous injection of ^Cy7^OMV-P or ^Cy7^OMV in mice bearing MB49 tumor. After 24 h, there was a stronger fluorescence signal in the tumor tissue in the ^Cy7^OMV-P group compared to the ^Cy7^OMV group (Fig. 2f and g). At different time points, mice were sacrificed immediately after in vivo imaging to harvest liver, kidney, spleen, tumor, and dLNs. We detected Cy7 fluorescence in the liver and kidney as early as 0.5 h, reflecting the circulation and accumulation of OMV, which continued to increase in liver, kidney, tumor, and dLNs over 12 h (Additional file 1: Figure S1c). The fluorescence signal in the tumor in the OMV-P group peaked at 24 h and decreased after 96 h, whereas the fluorescence signal in the OMV group almost disappeared (Fig. 2h). At each indicated time point, the Cy7 fluorescence signal in the OMV-P group was stronger than in the OMV group (Fig. 2i). In contrast, peptide-only FITC fluorescence decreased much faster, indicating OMV-P significantly increased peptide residence time in the tumor (Additional file 1: Figure S1d).

### OMV-P Suppressed lung cancer growth by upregulating cytotoxic T cell activity

**Fig. 3:**
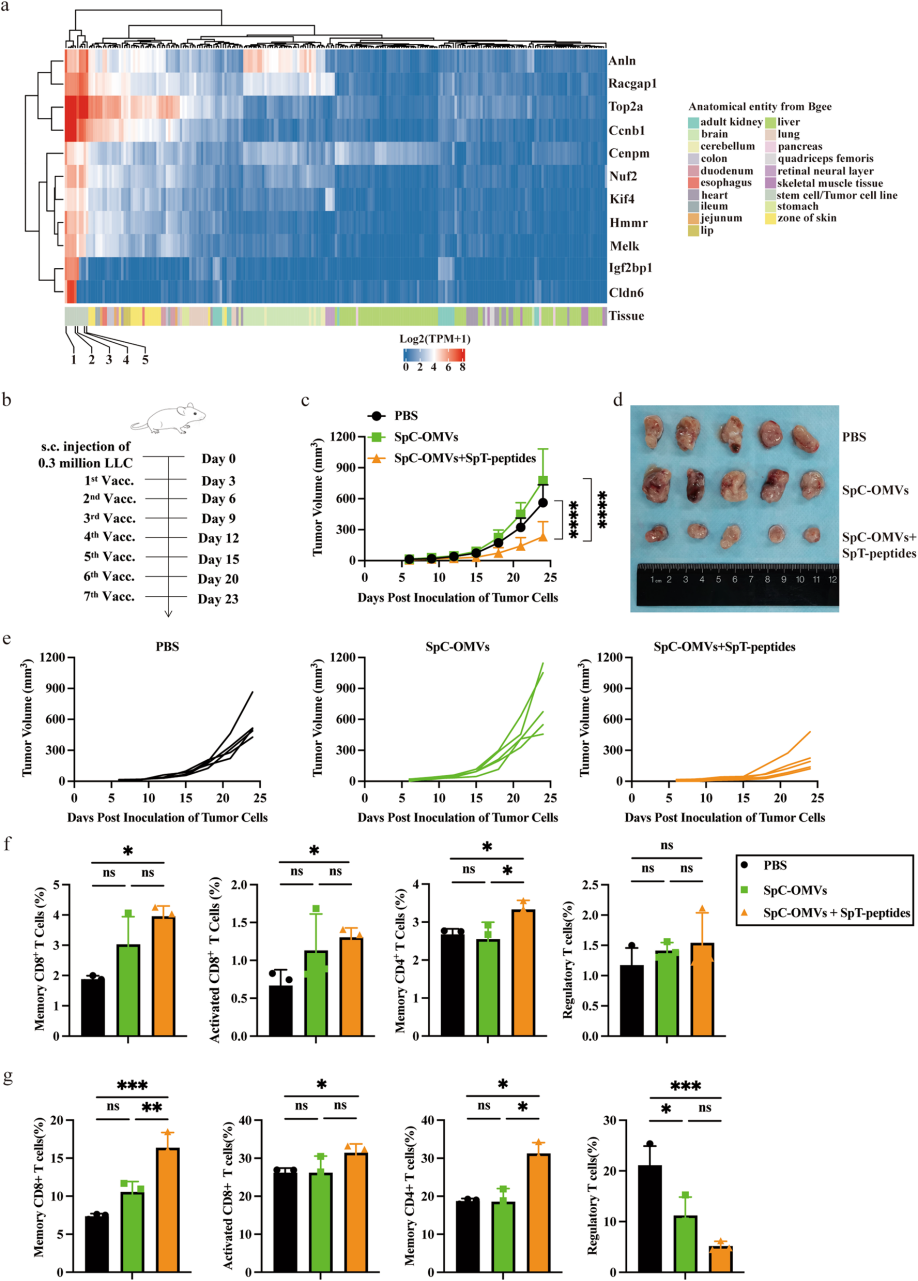

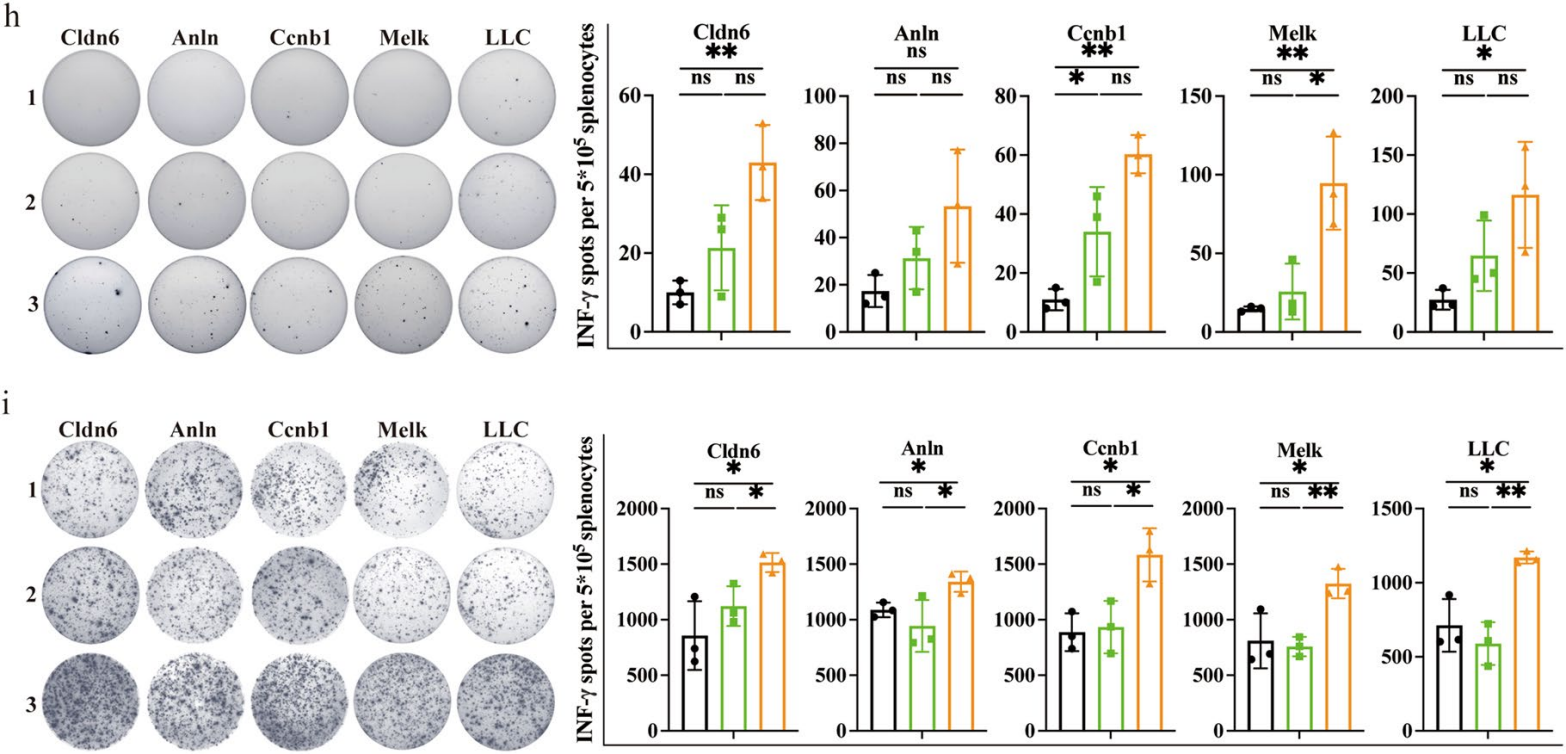
TAA-modified OMVs significantly suppressed lung cancer growth. **a** RNA sequencing revealed the expression profiles of 11 selected genes between mouse ESC and tumor cell lines compared to normal healthy tissues. 1. ESC (129); 2. ESC (C57BL/6); 3. Hepa1 − 6 cell; 4. LLC cell; 5. MB49 cell; **b** Schematic illustration examining how OMV-P inhibits lung cancer growth. C57BL/6 mice were s.c. injected with LLC cells in the right flank on day 0 and s.c. immunized on day 3, 6, 9, 12, 15, 20, and 23 for seven total vaccinations. **c**-**e** Antitumor effects of OMV-P. Tumor size was measured every 3 days. **f**, **g** Quantitative analysis of different subset of immune cells in lymph nodes **f** and PBMCs **g** by flow cytometry (n = 3). **h**, **i** Quantitative analysis of ELISPOT assay for IFNγ secretion to detect immune cell activation in splenocytes (**h**) and TILs (**i**) against selected epitopes and LLC tumor cells. 1, PBS. 2, SpC-OMVs. 3, SpC-OMVs + SpT-peptides. Data are shown as mean ± SD. One-way ANOVA with a Tukey multiple comparisons test or unpaired two-tailed Student’s t-test. NS, no significance; *, p < 0.05; **, p < 0.01; ***, p < 0.001; ****, p < 0.0001

To evaluate whether the tumor inhibitory effect of OMV-P can be applied to other tumors, we first examined if the selected peptides were specifically upregulated in different types of tumors. The RNA-seq results revealed high expression levels of 11 epitopes in ESCs, liver cancer cell line, bladder cancer cell line, and lung cancer cell line (Fig. 3a). Given that the antitumor effects of Cldn6, Anln, Ccnb1, and Melk have already been verified in bladder cancer, we used these four epitopes in our engineered OMVs. Mice bearing LLC cells were vaccinated with these OMV-Ps and tumor size was measured every 3 days (Fig. 3b). The results showed these OMV-Ps remarkably repressed lung cancer growth (Fig. 3c–e). Mice were sacrificed and blood and dLNs were collected to measure the ratio of different subsets of immune cells. Similar to the outcome obtained in the mouse bladder cancer model, the OMV-Ps resulted in elevated levels of CD8^+^ effector T cells, CD4^+^ memory T cells, and CD8^+^ memory T cells in PBMCs and dLNs in the mouse LLC model (Fig. 3f, g). In addition, we detected reduced levels of regulatory T cells (Treg) in the dLN of the OMV-P groups (Fig. 3g). Mice spleens and tumors were harvested and lymphocytes were extracted. Lymphocytes were cultivated on plates coated with interferon-γ (IFN-γ) capturing antibodies, with synthesized peptides and LLC cells as stimulants. The ELISPOT assay detected more IFN-γ-positive spots in the spleens and tumors of the OMV-P groups, indicating increased activation of T cells (Fig. 3h, i). These findings show that OMV-P inhibited lung cancer growth via promoting T cell-mediated immune responses.

### OMV Co-delivery of TAAs and PD-L1 antibody further inhibited tumor growth

**Fig. 4:**
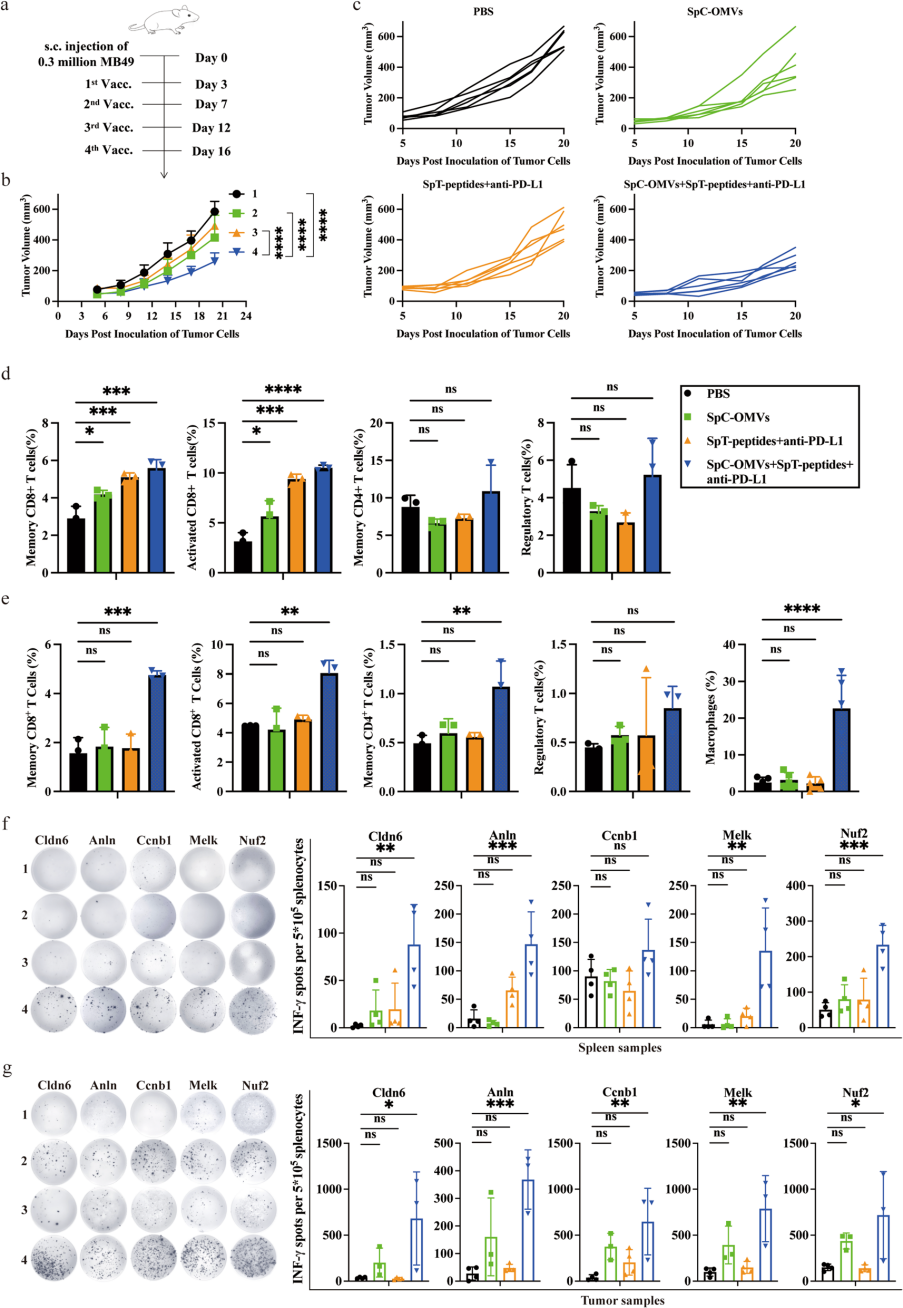
OMV co-delivery of both TAAs and PD-L1 antibody significantly inhibited tumor growth. **a** Schematic illustration examining how OMV-PP inhibits bladder cancer growth. C57BL/6 mice were s.c. injected with MB49 cells in the right flank on day 0 and s.c. immunized on day 3, 7, 12, and 16 for four total vaccinations. **b**, **c** Antitumor effect of OMV-PP. Tumor size was measured every 3 days. **d** Quantitative analysis of different subsets of CD3^+^CD45^+^ T cells in lymph nodes by flow cytometry (n = 3). e) Quantitative analysis of different subsets of immune cells in PBMCs by flow cytometry (n = 3 or 5). **f**, **g** Quantitative analysis of ELISPOT assay for IFNγ secretion to detect immune cell activation of splenocytes (**f**) and TILs (**g**) against selected epitopes. 1, PBS. 2, SpC-OMVs. 3, SpT-peptides + anti-PD-L1. 4, SpC-OMVs + SpT-peptides + anti-PD-L1. Data are shown as mean ± SD. One-way ANOVA with a Tukey multiple comparisons test or unpaired two-tailed Student’s t-test. NS, no significance; *, p < 0.05; **, p < 0.01; ***, p < 0.001; ****, p < 0.0001

The inhibitory tumor microenvironment is considered to be the main reason for the poor efficacy of cancer therapies. Given that PD-1/PD-L1 signaling has been shown to regulate T cells deactivation ^12^, we attached PD-L1 antibody onto the surface of OMVs to block PD-1/PD-L1 signaling to induce T cell-mediated immunity. Mice bearing MB49 tumors were immunized four times with the PD-L1 antibody-bound OMV-P (OMV-PP) and sacrificed on day 20 after tumor inoculation (Fig. 4a). Mice primed with OMV-PP showed a significant reduction in tumor size (Fig. 4b, c). Subsequent flow cytometry analysis revealed increased CD8^+^ effector T cells, CD4^+^ memory T cells, CD8^+^ memory T cells, and macrophages in PBMCs (Fig. 4d). Similarly, the ratio of CD8^+^ effector T cells and CD8^+^ memory T cells increased in dLNs-derived lymphocytes (Fig. 4e). We further performed ELISPOT with five peptides (MELK (Maternal Embryonic Leucine Zipper Kinase), ANLN (Anillin), CLDN6 (Cludin 6), NUF2, CCNB1 (Cyclin B1)) and measured IFN-γ secretion in lymphocytes isolated from spleens and tumors. Notably, T cell response against MELK, ANLN, CLDN6, or NUF2 was observed in the spleens of mice primed with OMV-PP (Fig. 4f). In tumors from the vaccinated group, all five peptides stimulated strong T cell response (Fig. 4g). The above data confirms the potential of OMV-PP on inhibiting tumor growth via prompting the activation of specific T cells.

### Mechanism of OMV-PP-induced antitumor effect

**Fig. 5:**
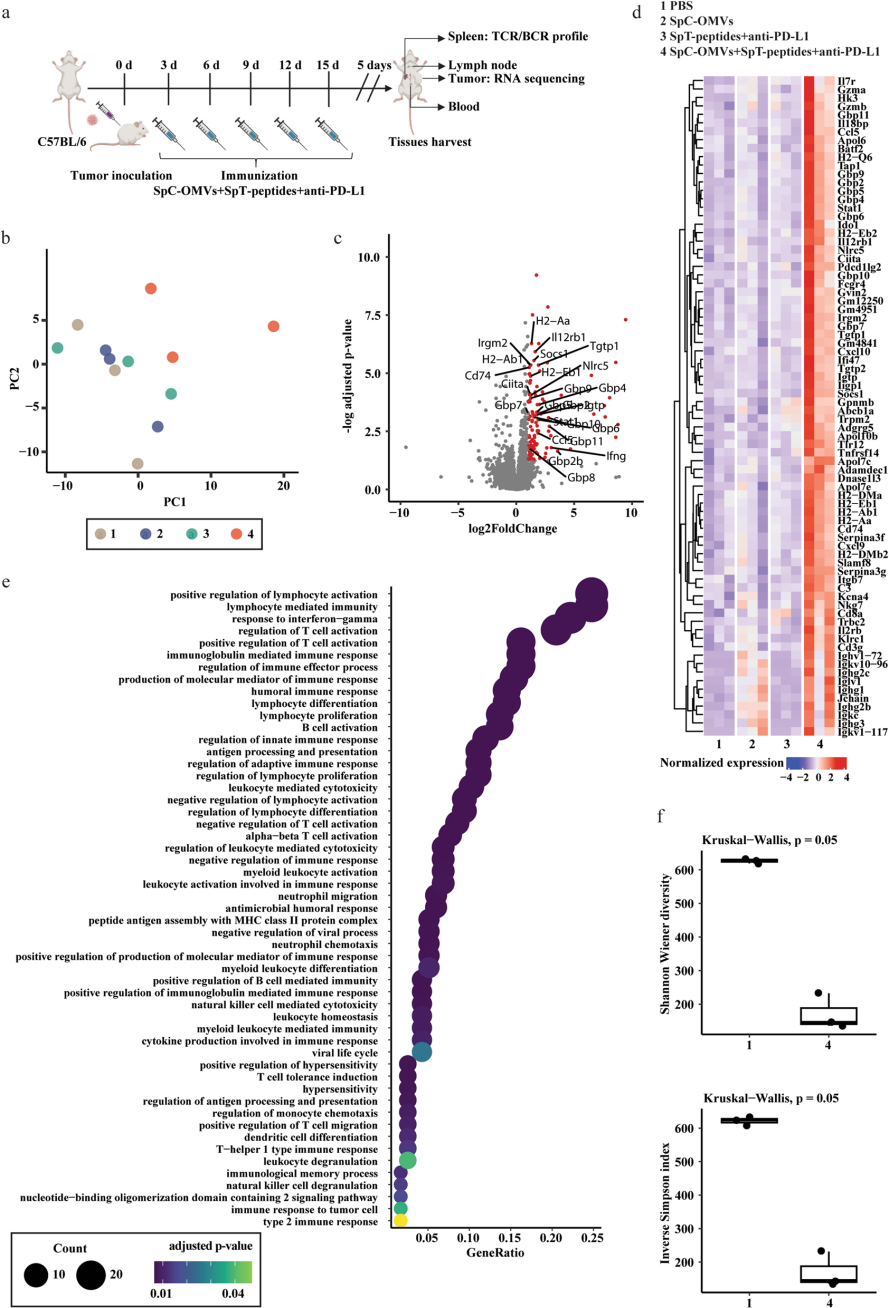
**a** Schematic illustration examining how OMV-PP inhibits bladder cancer growth. C57BL/6 mice were s.c. injected with MB49 cells in the right flank on day 0 and s.c. immunized on day 3, 6, 9, 12, and 15 for five total vaccinations. Mice were sacrificed 5 days after the final vaccination organ harvest. **b** Principal component analysis of RNA-Seq data. **c** Plots showing differentially expressed genes in group 4 compared to group 1. Dots in red indicate genes significantly upregulated in group 4. Significantly upregulated genes belonging to INF-γ response are labeled. **d** Heatmap plot showing the expression levels of upregulated genes in different groups. **e** Enriched gene ontology (GO) terms of significantly upregulated genes. **f** TCR-β repertoire diversities across groups 1 and 4. 1, PBS. 2, SpC-OMVs. 3, SpT-peptides + anti-PD-L1. 4, SpC-OMVs + SpT-peptides + anti-PD-L1

To further understand the mechanism underlying the inhibition of tumor growth by OMV-PP, we collected tissues from mice bearing MB49 tumors after treatment with OMV-PP (Fig. 5a). The harvested tumors were immediately immersed in liquid nitrogen and stored at -80 °C. The samples were then processed for RNA sequencing. Principal component analysis showed there were distinct separations among PBS, OMV, Peptides + PD-L1 antibody (PP), and OMV-PP groups, indicating a high level of similarity among the biological replicates from different samples (Fig. 5b). Interestingly, the volcano plot indicated variably expressed genes between the OMV-PP and other groups, for example, OMV-PP groups had increased expression levels of Irgm2 and Il12rb1, which are involved in the IFN-γ response (Fig. 5c). Genes involved in CD4^+^ T cells, CD8^+^ T cells, and DCs were also enriched in OMV-PP groups (Additional file 2: Figure S2). The gene ontology (GO) analysis showed 78 enriched genes (Fig. 5d) with a total of 53 GO terms, including the regulation of Th1 immune response, positive regulation of T cell activation, antigen processing and presentation, regulation of leukocyte-mediated cytotoxicity, response to IFN − γ, etc. (Fig. 5e). Given that IFN-γ-dependent T cell immunity is vital for tumor eradication, we performed RNA sequencing on total RNA from mouse splenocytes to examine the immune repertoire. The repertoire analysis showed lower T cell receptor (TCR)-β repertoire diversity in the OMV-PP group compared to the other groups, indicating the enhancement of specific TCR clones. This suggests the antitumor effects of OMV-PP are associated with enhanced T cell immunity.

### OMV-PP strongly induced antitumor T cell immunity

**Fig. 6:**
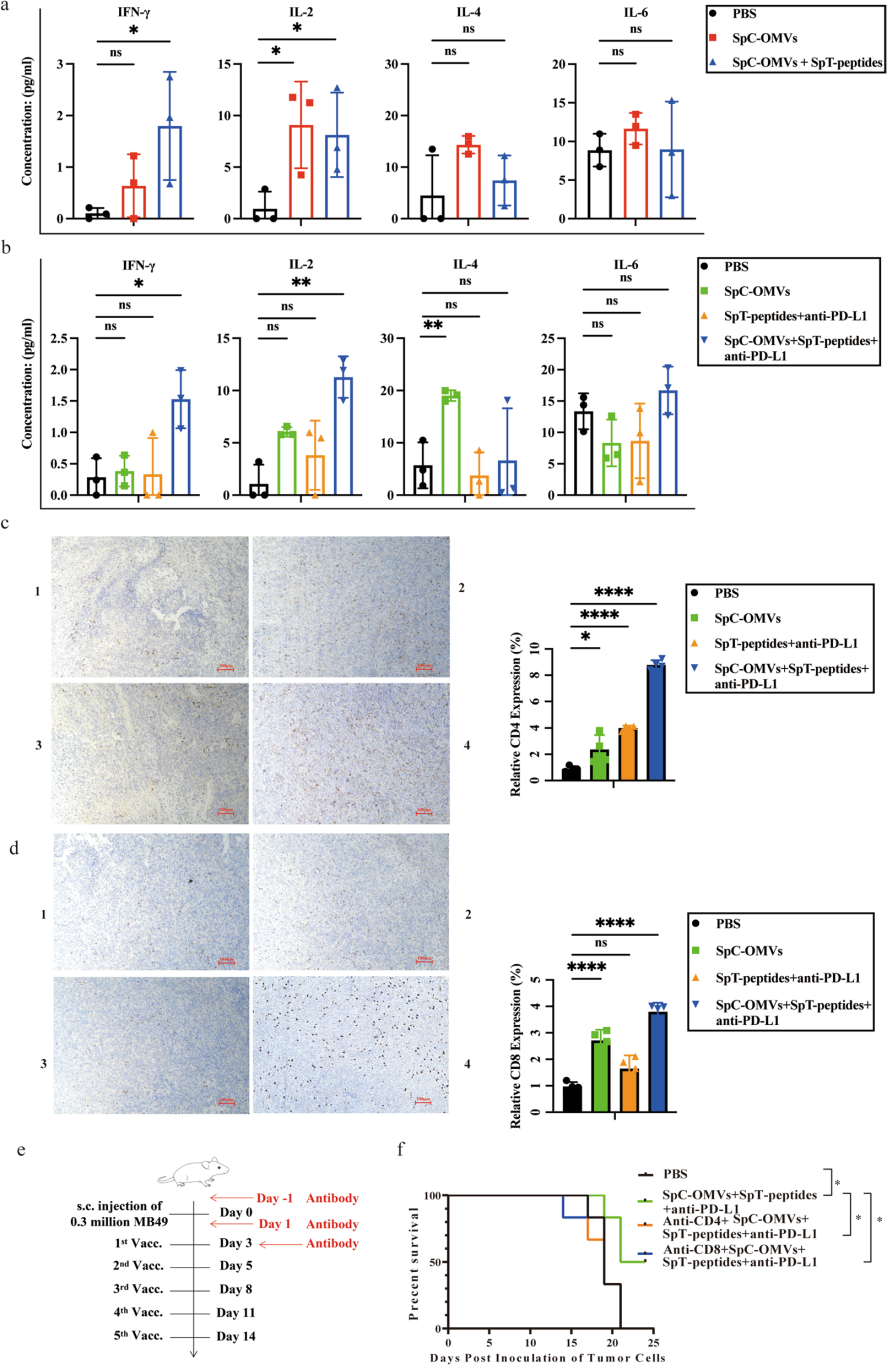
The antitumor effects of OMV-PP were T cell immunity-dependent. a, b) Multiplex cytokine assay was performed to determine the expression profiles of serum IL-2, IL-4, -IL-6, and INF-γ in lung cancer model (**a**) and bladder cancer model (**b**). **c**, **d** IHC images showing the infiltration level of CD4^+^ and CD8^+^ T cells in tumor tissues. 1, PBS. 2, SpC-OMVs. 3, SpT-peptides + anti-PD-L1. 4, SpC-OMVs + SpT-peptides + anti-PD-L1. Scale bar: 100 μm. Quantitative analysis on the right. Data are shown as mean ± SD. **e** Schematic illustration determining whether CD4^+^ and CD8^+^ T cells are integral for the antitumor effects of OMV-PP. C57BL/6 mice were s.c. inoculated with MB49 cells in the right flank on day 0 and s.c. immunized on day 3, 5, 8, 11 and 14 for five total vaccinations. From day -1, 1, and 3 after tumor inoculation, 10 mg kg^−1^ of CD8^+^ T cell depletion antibody and 10 mg kg^−1^ of CD4^+^ T cell depletion antibody were injected intraperitoneally in mice. **f** Survival time of MB49 tumor-bearing mice were measured. One-way ANOVA with a Tukey multiple comparisons test or unpaired two-tailed Student’s t-test. *NS* no significance; *, p < 0.05; **, p < 0.01; ***, p < 0.001; ****, p < 0.0001

To confirm the mechanism of the inhibitory effect of OMV-PP on tumor growth, we tested various secreted cytokines in the serum. Analysis of blood derived from the mice inoculated with LLC cells showed OMV-P elevated serum levels of IFN-γ and interleukin-2 (IL-2), but did not have much effect on IL-4 and IL-6 compared with the other three groups (Fig. 6a). In the mouse bladder cancer model, OMV-PP induced increased serum levels of IFN-γ and IL-2 (Fig. 6b). Similarly, OMV-PP significantly altered serum levels of IL-4 and IL-6, whereas OMV alone only affected IL-4 (Fig. 6b). In the tumor tissues, OMV-PP resulted in the increased infiltration of CD4^+^ and CD8^+^ T cells (Fig. 6c and d). Consistently, the cytotoxic effects of T cells and helper T cells were confirmed by CD8 depletion and CD4 depletion, respectively. Depletion of CD8^+^ and CD4^+^ T cells abrogated the antitumor effects of OMV-PP, resulting in reduced survival similar to that of untreated mice (Fig. 6f). The above findings confirmed that the tumor inhibition effects of OMV-PP were via the enhancement of antitumor T cell immunity.

## Discussion

Epitopes from iPSC and ESC represent a large source of candidates for the development of cancer vaccines. Currently, a wide range of vaccines based on ESCs-derived oncofetal antigens are being tested in pre-clinical studies for their potential in inhibiting tumor growth [32–34], while some single-epitope vaccines have already reached stage I/II clinical trials [35]. In our previous study, we reported that TAAs shared by both ESCs and tumor cells could potently trigger an immune response against bladder cancer [1]. However, peptide-based vaccines have issues of short half-life of peptides and short-lived antitumor immune response in vivo [36]. To extend the half-life and tumor-targeting ability of selected TAAs, we employed OMVs as a platform for the delivery of synthesized epitopes. We first constructed an engineered SpA strain Δ*lpxM*Δ*pal* to reduce the toxicity of OMVs and to increase its efficiency. Next, OmpA, a component of the bacterial cell outer membrane, was fused with SpyCatcher for linking SpyTag-attached peptides. To counteract the immune suppressive microenvironment of the tumor, we also altered the surface of OMVs to allow delivery of a PD-L1 antibody to block PD-1-mediated deactivation of T cells. Our engineered OMVs on the SpA surface were able to bind both TAAs and PD-L1 antibody efficiently (Fig. 1f).

To confirm the efficacy of our delivery system, we injected our engineered OMVs into mice bearing MB49 tumors. The biodistribution of OMV-P or OMVs at different time points was monitored by in vivo imaging. The results showed that OMV-P was able to infiltrate more into the tumor tissues than OMVs, as seen by the fluorescence signal remaining in the tumors in the OMV-P group, whereas the signal completely disappeared 4 days after vaccination in the OMV group. Moreover, mice immunized with FITC-labeled epitopes showed the synthesized peptides were hardly detected in the tumors on day 2 after immunization (Fig. 2g), indicating the engineered OMVs allow greater retention of epitopes and together with TAAs enhances targeting to the tumor. While the bulk of OMV-P was observed in the liver and kidneys, some fluorescence signal was also detected in the spleen 12 h after immunization (Fig. 2g). It is therefore possible that the delivered epitopes could trigger an immune response in the spleen. We showed that OMV-P significantly decreased tumor size in both the bladder cancer and lung cancer models. In agreement with the results from our previous research [1], the flow cytometry analysis revealed elevated ratios of CD8^+^ effector T cells, CD4^+^ memory T cells, and CD8.^+^ memory T cells in OMV-P-treated mice, which suggests ESC-based epitopes have the potential to inhibit different types of tumor growth via inducing antitumor T cell responses. The ELISPOT assay also showed the OMV-delivered epitopes enhanced specific T cell responses. The T cell types are critical for the generation and maintenance of long-term memory and recall responses in vaccinated animals [37]

We engineered our OMV system to co-deliver PD-L1 antibody to block the immunosuppressive PD-L1 pathway and enhance the tumor-targeting of TAAs to activate CD8^+^ T cells. The OMV-PP system demonstrated enhanced efficacy for the two immunotherapies. To improve the limitations of anti-PD-1/PD-L1 treatment, strategies have been proposed to target different steps including enhancing T cell priming by increasing antigen presentation, increasing T cell infiltration, and increasing the insufficient concentration of PD-1/PD-L1 antibody in the tumor to reverse the immunosuppressive microenvironment. Our new co-delivery system was able to increase the level of PD-L1 antibody in the tumor compared to in other parts of the body. The data showed that OMV-PP combining both anti-PD-L1 and TAAs in a single platform was more effective in inhibiting tumor growth than equivalent amounts of soluble anti-PD-L1 and TAAs or OMV alone. The OMV-PP treatment also increased levels of CD8^+^ effector T cells and CD8^+^ memory T cells, and induced IFN-γ-related immunity. To investigate the underlying mechanism of the inhibitory effects of OMV-PP, we next performed RNA sequencing of tumor tissues and TCR sequencing of the spleen. The RNA-seq findings showed the OMV-PP group had different gene clusters compared with the other groups, indicating the upregulation of specific genes. The majority of upregulated genes in the OMV-PP group were enriched in the activation of T cells and the INF-γ signaling pathways (Fig. 5d and e), indicating the activation of these signaling pathways was crucial for the antitumor immune response of OMV-PP. Antigen-specific T cells targeting ESC-based TAAs were significantly increased in both the tumor and spleen of the OMV-PP group (Fig. 4f and g). The TCR-seq findings showed a decreasing trend in the diversity of TCR clones in the OMV-PP group, indicating the expansion of specific TCR clones (Fig. 5f). As the duration of tumor treatment increased, the diversity of TCR clones gradually decreased [38]. To validate these results, we further examined the secretion of cytokines in the serum and the infiltration of T cells in tumors. The OMV-PP treatment increased serum IFN-γ and IL-2, which are both secreted by activated T cells or other immune cells. Moreover, the IHC imaging revealed OMV-PP increased infiltration levels of CD4^+^ and CD8^+^ T cells into the tumor tissues (Fig. 6). These results indicate that the antitumor effect induced by OMV-PP involves T cell immune responses, which was confirmed by the antibody depletion tests. In vivo deletion of T lymphocyte subsets including CD4^+^ and CD8^+^ T cells reversed the OMV-PP-induced repression of cancer growth. The above results indicate that the OMV co-delivery of TAAs and anti-PD-L1 can effectively induce T cell infiltration and specific T cell expansion in tumors, as well as induce the formation of memory T cells in the spleen, which together contribute to the antitumor effects.

**Fig. 7:**
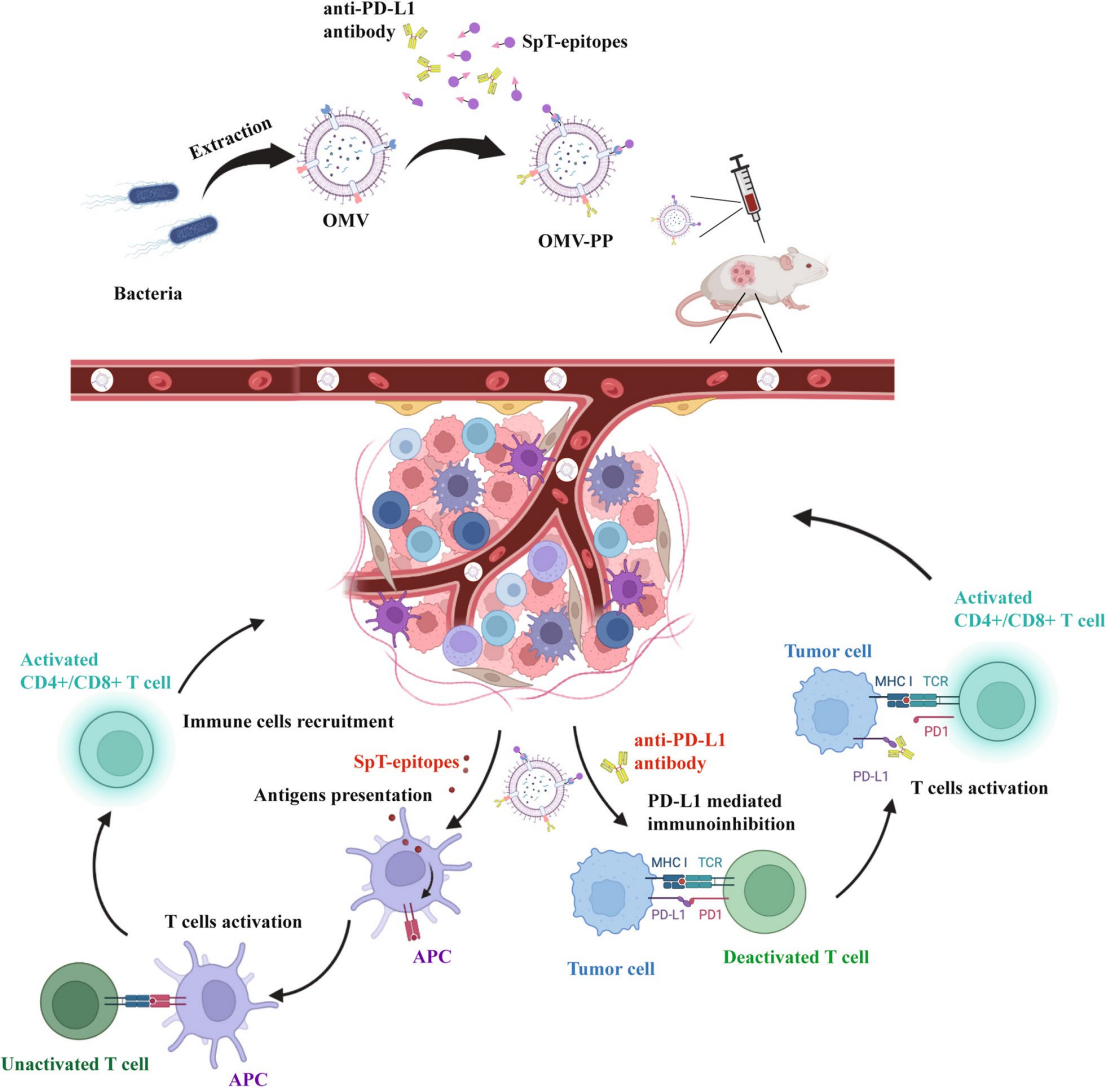
Scheme illustration of the OMV delivery of TAAs and PD-L1 antibodies targeting tumors. Mice were s.c. injected with OMVs conjugated with both SpT-epitopes and PD-L1 antibodies. After being recognized by APCs, the selected ESC-based epitopes induced the activation of T cells, triggering the enrichment of activated immune cells in tumor tissues, while the delivery of PD-L1 antibody blocked PD-1 signaling to rescue the deactivated T cells, leading to effective specific T cell immunity against the tumor

In conclusion, we constructed a new delivery system using engineered OMVs that efficiently combine TAAs and PD-L1 antibodies for targeting the tumor and specific organs. The OMV-PP can activate antitumor T cells, induce the expansion of specific T cells, relieve the immunosuppressive environment of the tumor, and enhance antitumor T cell responses. Our novel co-delivery system could lead to the effective delivery of personalized tumor vaccines or universal tumor vaccines and other clinical applications (Fig. 7).

### Supplementary Information

**Additional file 1**: **Figure S1**. a) Western blotting to detect the expression of OmpA and His-tag on engineered OMVs. b) Representative TEM image showing no observable difference between OMVs and OMV-PP. Scale bar: 200 nm. OMV-PP, OMVs binding both SpT-peptides and PD-L1 antibodies. c) Cy7-labeled OMVs were used for the IVIS analysis. Mice bearing MB49 cells were s.c. immunized and sacrificed at different time points to show the in vivo distribution of OMVs or OMV-P. Schematic illustrations of organs on the left. At 12 h post-injection, Cy7-labeled OMVs were detected in lymph nodes and tumors in both groups. On day 1, the OMV fluorescence signal in the tumor disappeared, whereas OMV-P signals remained in the tumors. d, day. d) mice bearing bladder cancer were injected with FITC-labeled peptides. At different time points, mice were sacrificed for organ harvesting to detect in vivo distribution of peptides. schematic illustrations of organs on the left. On day 1, peptides were observed to target the tumors. Peptides quickly degraded and vanished on day 2. d, day.

**Additional file 2: Figure S2**. Cell type enrichment analysis of the RNA-seq data with function scores for each cell type in different study groups.

## Data Availability

The datasets generated during and/or analyzed during the current study are publicly available.

## Abbreviations

PD-L1: Programmed cell death 1 ligand 1
APC: Antigen-presenting cells
SpC: SpyCatcher
SpT: SpyTag
SpA: Staphylococcal Protein A
OMV: Outer membrane vesicles
OMV-P: OMVs binding SpyTag-peptide
OMV-PP: OMV-P binding SpyTag-peptide and anti-PD-L1 antibody
CD4: Cluster of differentiation 4
CD8: Cluster of differentiation 8
IgG: Immunoglobulin
TAA: Tumor-associated antigen
ESC: Embryonic stem cell
PBMC: Peripheral blood mononuclear cell
dLN: Drain lymph node
IL: Interleukins
IFN-γ: Interferon gamma
TCR: T cell receptor
BCR: B cell receptor
