# Supplementary figures and images for: Enhancing immune responses of ESC-based TAA cancer vaccines with a novel OMV delivery system
Source: J Nanobiotechnology. 2024 Jan 3;22:15. doi: 10.1186/s12951-023-02273-8 (PMC10763241; doi:10.1186/s12951-023-02273-8)

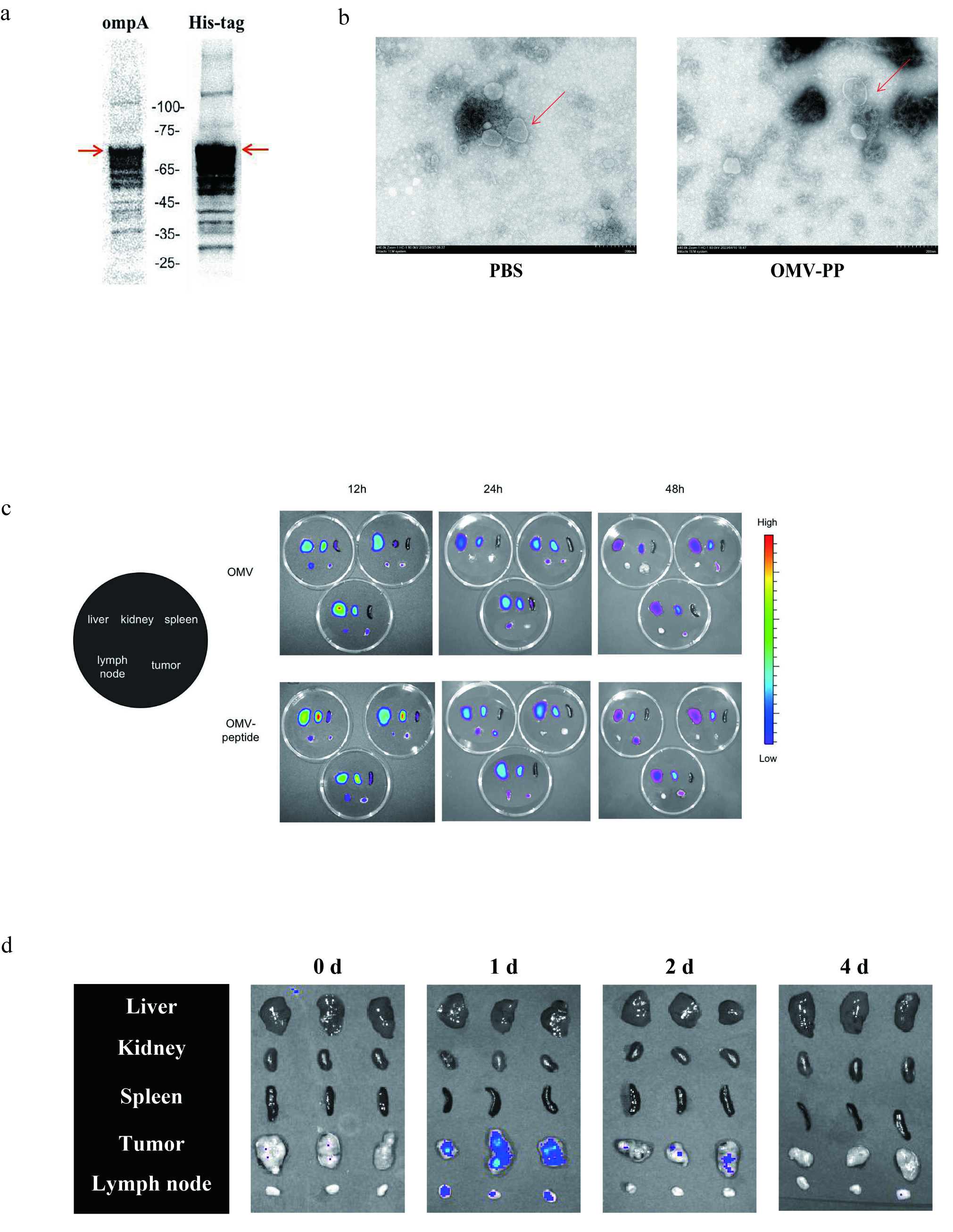

Supplement: Supplementary file 1 — Additional file 1: Figure S1. a) Western blotting to detect the expression of OmpA and His-tag on engineered OMVs. b) Representative TEM image showing no observable difference between OMVs and OMV-PP. Scale bar: 200 nm. OMV-PP, OMVs binding both SpT-peptides and PD-L1 antibodies. c) Cy7-labeled OMVs were used for the IVIS analysis. Mice bearing MB49 cells were s.c. immunized and sacrificed at different time points to show the in vivo distribution of OMVs or OMV-P. Schematic illustrations of organs on the left. At 12 h post-injection, Cy7-labeled OMVs were detected in lymph nodes and tumors in both groups. On day 1, the OMV fluorescence signal in the tumor disappeared, whereas OMV-P signals remained in the tumors. d, day. d) mice bearing bladder cancer were injected with FITC-labeled peptides. At different time points, mice were sacrificed for organ harvesting to detect in vivo distribution of peptides. schematic illustrations of organs on the left. On day 1, peptides were observed to target the tumors. Peptides quickly degraded and vanished on day 2. d, day. [file 12951_2023_2273_MOESM1_ESM.tif]

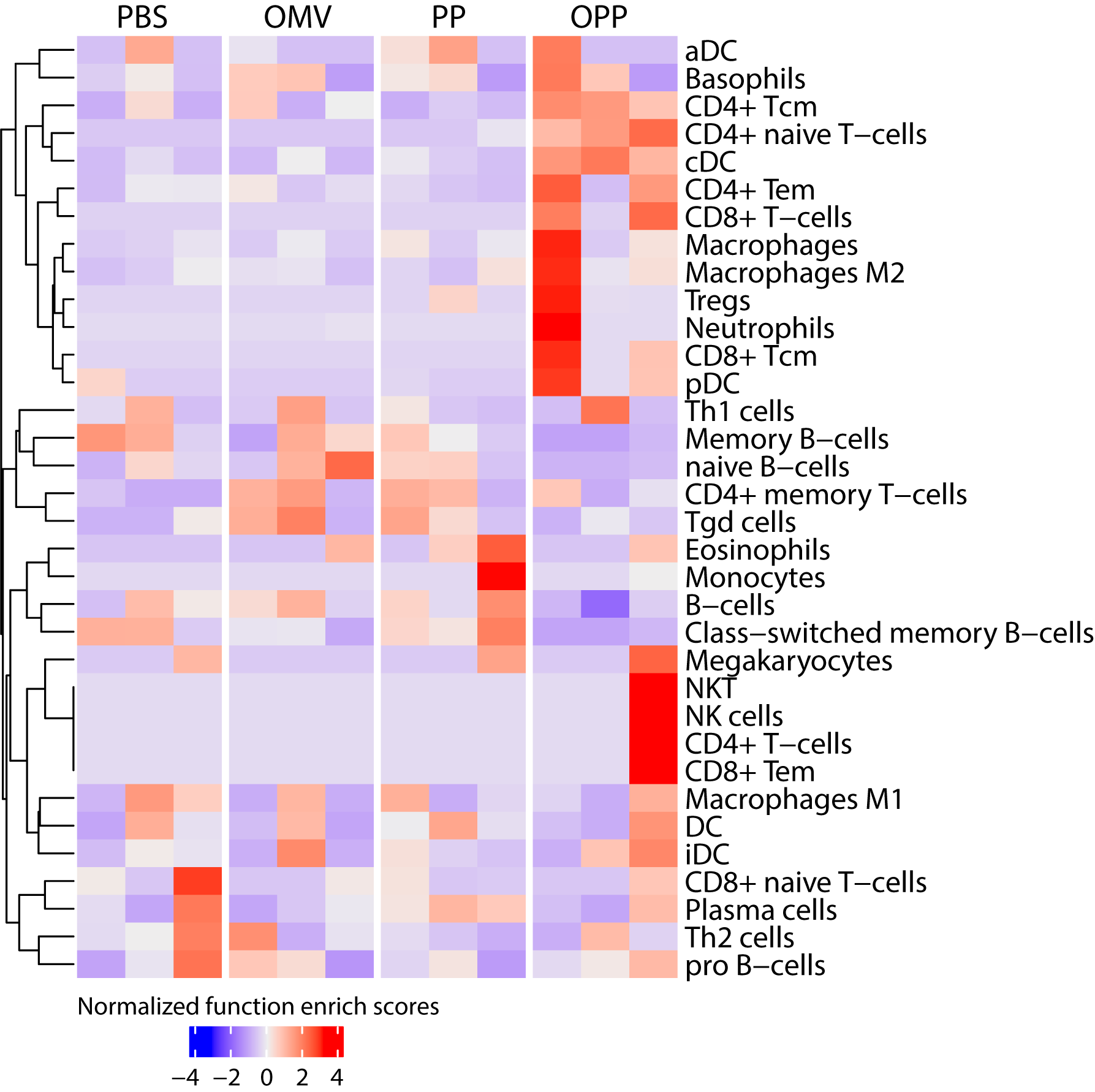

Supplement: Supplementary file 2 — Additional file 2: Figure S2. Cell type enrichment analysis of the RNA-seq data with function scores for each cell type in different study groups. [file 12951_2023_2273_MOESM2_ESM.tif]
